# The Disparities in Patient Portal Use Among Patients With Rheumatic and Musculoskeletal Diseases: Retrospective Cross-sectional Study

**DOI:** 10.2196/38802

**Published:** 2022-08-31

**Authors:** Enid Y Sun, Carolina Alvarez, Leigh F Callahan, Saira Z Sheikh

**Affiliations:** 1 Section of Rheumatology Department of Medicine Temple Lewis Katz School of Medicine Philadelphia, PA United States; 2 Thurston Arthritis Research Center University of North Carolina Chapel Hill, NC United States; 3 Division of Rheumatology, Allergy, and Immunology Department of Medicine University of North Carolina School of Medicine Chapel Hill, NC United States

**Keywords:** COVID-19, telemedicine, telehealth, health technology, health care disparities, patient portal, rheumatology, musculoskeletal diseases, chronic disease, digital health, MyChart, rural area, minority population, virtual care

## Abstract

**Background:**

During the COVID-19 pandemic, the shift to virtual care became essential for the continued care of patients. Individuals with rheumatic and musculoskeletal diseases (RMDs) especially require frequent provider visits and close monitoring. To date, there have been limited studies examining inequities in health technology use among patients with RMDs.

**Objective:**

Our goal was to identify characteristics associated with patient portal use before and after the COVID-19 pandemic in a convenience sample of patients with RMDs from a large academic medical center.

**Methods:**

In this cross-sectional study, Epic electronic medical record data were queried to identify established patients of the University of North Carolina Hospitals adult rheumatology clinic between November 1, 2017, through November 30, 2019. Demographic and clinical data were collected to compare MyChart (Epic’s patient portal) users with nonusers before and after the COVID-19 pandemic. MyChart activation and use were modeled using logistic regression and adjusted odds ratios, and confidence intervals were estimated.

**Results:**

We identified 5075 established patients with RMDs who met the inclusion criteria. Prior to the pandemic, we found that younger age (*P*<.001), suburban residence (*P*=.05), commercial/state insurance (*P*<.001), military insurance (*P*=.05), and median income >US $50,000 (*P*<.001) were associated with significantly higher odds of MyChart activation. Male sex (*P*<.001), being of Black or African American (*P*<.001) or “other” race (*P*<.001), Spanish as a primary language (*P*<.001), rural residence (*P*=.007), Medicaid insurance (*P*<.001), and median income of <US $25,000 (*P*=.01) were associated with lower odds of MyChart activation. Following COVID-19, younger age (*P*<.001), commercial insurance (*P*=.03), state insurance (*P*=.02), and median income of US $50,000-75,000 (*P*=.01) were associated with significantly higher odds of MyChart use. However, being of Black or African American (*P*<.001) or “other” race (*P*=.01), Spanish as a primary language (*P*=.002), male sex (*P*=.004), rural residence (*P*=.005), and having no insurance (*P*<.001) or Medicaid (*P*=.008) were associated with lower odds of MyChart use.

**Conclusions:**

Residence in a rural area, being of minority race/ethnicity, older age, male sex, lower median income, Medicaid, being uninsured, and non-English primary language are associated with lower odds of patient portal activation and use. Future health policy and clinical practice measures should focus on reducing barriers to health technology adoption among these groups.

## Introduction

Rheumatic and musculoskeletal diseases (RMDs) are complex chronic conditions that require lifelong care. Patients may experience flares or acute complications related to their disease, requiring close communication with their rheumatologist. Patients with these conditions often take medications that require frequent monitoring and irregular dosing schedules. These aspects of RMD management require a high level of patient agency and open avenues for patient-provider contact and communication. Digital technology such as patient portals, health apps, and wearable technologies allow patients to manage and participate in their own care [1].

Many studies have shown positive effects on patient outcomes and satisfaction when patients are engaged in their own care through digital technologies [1-4]. In a systematic review by de Jong et al [2], patients who were able to communicate with their physicians had increased knowledge and self-management regarding their chronic condition, decreased health care visits, and improved psychosocial and clinical outcomes. In another study of patients with rheumatoid arthritis, patients who received weekly SMS text messages had better medication adherence than patients who did not receive the SMS text messaging intervention [3]. Participation in a web-based arthritis self-management program was associated with improved health status measures and self-efficacy in a study of patients with RMDs (ie, rheumatoid arthritis, osteoarthritis, or fibromyalgia) [4].

Health technology use became a necessity in early 2020 following the SARS-CoV-2 (COVID-19) outbreak, which forced health care systems around the world to adapt in the face of uncertainty. During this period, there has been a large shift to virtual care. Although this change has the possibility to close the gap in health care delivery in the United States, studies have shown that there are disparities in health technology use and the use of technology in general [5-11]. These studies have shown that low health literacy, lower educational attainment, residence in a rural area, being of minority race/ethnicity, and older age are associated with lower rates of health app and general technology use (ie, computer and cellphone ownership) [5-11].

To date, there have been limited studies examining inequities in health technology use among patients with rheumatologic conditions, and to our knowledge, none have looked at how COVID-19 has affected the patterns of health technology use among this patient population. Our goal was to identify the characteristics of patient portal users versus nonusers from a group of patients at a large hospital-based rheumatology clinic. We aimed to identify disparities and potential barriers to telehealth adoption among patients with rheumatologic conditions to help close the gap in health technology use.

## Methods

### Study Subjects

In this cross-sectional study, Epic electronic medical record data were queried to identify established patients of the University of North Carolina Hospitals (UNCH) adult rheumatology clinic between November 1, 2017, through November 30, 2019. “Established” patients were defined as patients who had at least one return visit during the 2-year study period. We specifically excluded “new” patients since these individuals may be seen for 1 consultative visit without further follow-up in the UNCH system. We felt that including these subjects could underestimate patient portal activation or use among our population.

### Variables

Demographic and clinical data were collected from patient- and provider-entered information on Epic and used to compare the patients who activated Epic’s patient portal (MyChart) to patients who did not activate MyChart at the time of the initial data acquisition. Additional data on MyChart usage were collected for the following year to compare MyChart use 8 months prior to the start of telemedicine visits at our clinic (from July 1, 2019, to March 30, 2020; “prepandemic”) to the 8 months following the clinic’s adoption of virtual care (from April 1, 2020, to December 2, 2020; “postpandemic”). MyChart “activation” indicates that the patient, or a patient proxy, has enrolled for patient portal access. MyChart “usage” was defined as the patient or patient-assigned proxy using MyChart to read or send patient-provider messages or manage appointments.

Demographic information collected included age, sex, race or ethnicity as documented in the electronic medical record (American Indian or Alaska Native or Native Hawaiian or Pacific Islander, Asian, Black or African American, Hispanic or Latino, White, or “other” race), primary language (English, Spanish, or “other”), zip code and county of primary residence, and primary insurance payor. Patients were grouped into generational categories based on age at the time of initial data collection (November 2019): born from 1997 to the present (“Gen Z,” ages 17-22 years), born from 1981-96 (“Millennials,” ages 23-38 years), born from 1965-80 (“Gen X,” ages 39-54 years), born from 1946-64 (“Baby Boomers,” ages 55-73 years), and born from 1928-45 (“Silent Generation,” ages 74-91 years) [12]. North Carolina (NC) “rural,” “suburban,” and “urban” county designations were defined as average population densities of ≤250 people/square mile, 250-750 people/square mile, or ≥750 people/square mile, respectively, based on densities as reported in 2014 US Census population estimates [13]. Using individual income 2017 zip code data for NC from the Internal Revenue Service (IRS), we used the median gross income of each zip code to estimate individuals’ annual median gross income. Estimated median adjusted gross income was grouped in quartiles as reported in the IRS data [14]. Income data were not available for all zip codes; income data was not reported for zip codes with a low number of returns or in cases of nonresidential zip codes [14]. Thus, patients with NC zip codes without income information and patients with out-of-state zip codes were excluded from analysis. The clinical data collected included the most recent outpatient visit date and number of clinic visits (≤2 vs ≥3) within the study period.

### Statistical Analysis

Descriptive statistics were used to summarize study subjects and relevant variables. Counts and percentages were produced for categorical variables, whereas mean (SD) or median (IQR) were computed for continuous variables. Multivariable logistic regressions were separately modeled for the log odds of MyChart activation pre–COVID-19 (Model 1), MyChart use pre–COVID-19 and post–COVID-19 (Model 2), and MyChart use post–COVID-19 among nonusers pre–COVID-19 (Model 3). Model 2 used generalized estimating equations to account for the correlation between a patient’s pre–COVID-19 and post–COVID-19 MyChart use and the interaction of pre–COVID-19 or post–COVID-19 time, with all covariables tested and retained if *P*<.05; otherwise, overall effects were shown for this model.

**Figure 1:**
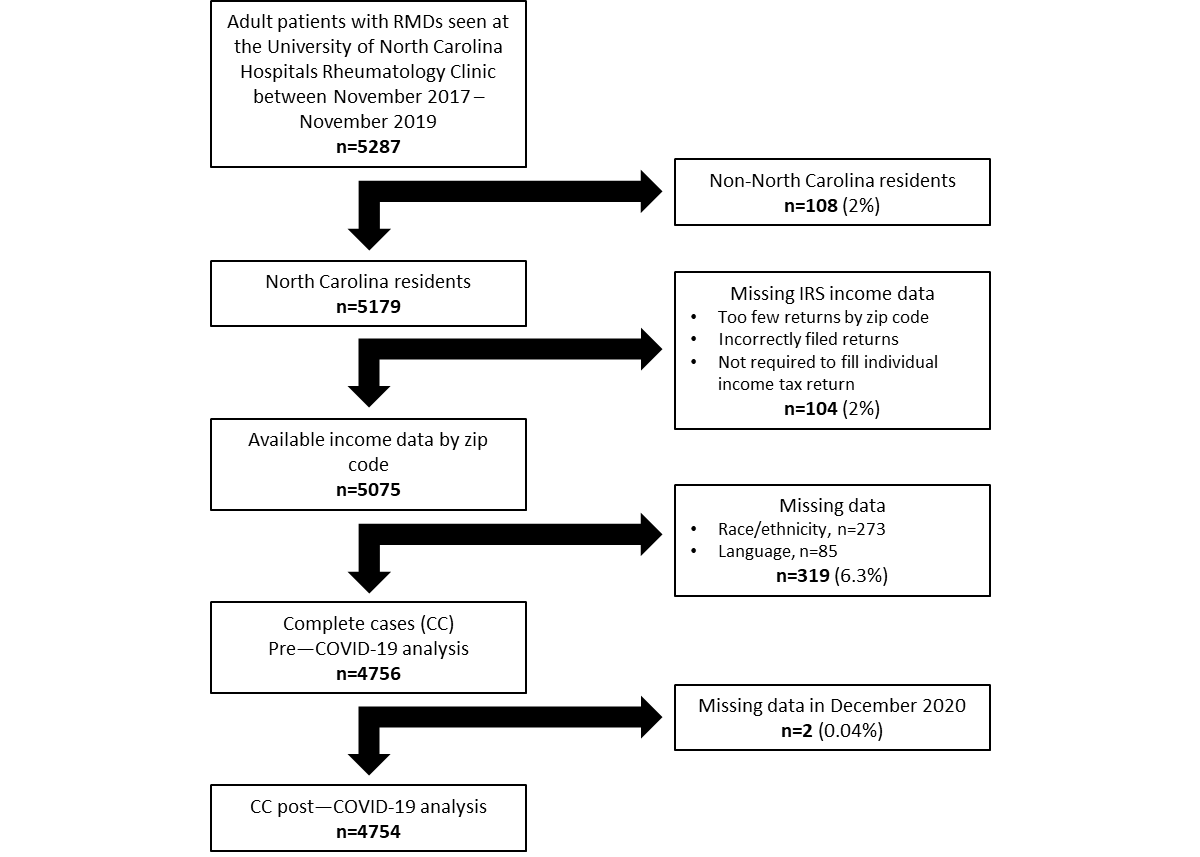
Subject inclusion and exclusion. IRS: Internal Revenue Service; RMD: rheumatic and musculoskeletal disease.

Models included all previously defined variables: visit date, the number of visits, age group, sex, race or ethnicity, primary language, county of residence, insurance, and median zip code–based income to produce adjusted odds ratios (aORs) and 95% CI. Complete cases were included for multivariable analyses, excluding subjects with missing variable items given that missing data rates (319/5075, 6.3%) were well below <10% (Figure 1) among NC residents with available IRS income by zip code.

Sensitivity analyses using multiple imputations of variables with missing information (race or ethnicity and primary language) were performed to assess the consistency of results. These variables were imputed using logistic regression by fully conditional specification methods for binary variables, which performs best for missing-at-random patterns and a missing proportion of less than 50%, to generate 10 imputed data sets for analyses [15]. Due to the exploratory nature of our study, corrections of statistical significance level were not performed [16]. All analyses were performed with SAS statistical software (version 9.4; SAS Institute Inc). Statistical significance was determined at *P*=.05.

### Ethics Approval

Our study was reviewed and approved by the University of North Carolina’s Institutional Review Board (protocol 19-3155) and adheres to the ethical principles of the Declaration of Helsinki. A waiver of informed consent was obtained due to the retrospective nature of our study.

## Results

### General Characteristics

We identified 5287 established patients who were seen at the UNCH rheumatology clinic during our study period, of whom 5075 patients were NC residents with available income data based on zip code. There were 4756 complete cases included in the pre–COVID-19 MyChart activation analyses and 4754 in post–COVID-19 MyChart use analyses (Figure 1).

Descriptive statistics for key characteristics are shown for the cohort of NC residents with income data (N=5075; Table 1). The mean age of the NC cohort was 54.7 (SD 15.4) years, and 73.9% (n=3749) were female. Of the 5075 established patients, 51% (n=2586) identified as White and 26.4% (n=1342) identified as Black or African American. In all, 88% (n=4478) identified English as their primary language, whereas 9.2% (n=469) reported Spanish as their primary language. Patients were almost evenly split between urban (n=1679, 33.1%), suburban (n=1506, 29.7%), and rural (n=1890, 37.2%) residences. Over two-thirds (n=3563, 70.2%) had a median adjusted gross income between US $25,000 to <US $50,000. Regarding insurance, 37% (n=1851) had Medicare, 25.1% (n=1276) had commercial insurance, 10.1% (n=512) had Medicaid, and 16.9% (n=859) were uninsured.

We examined MyChart activation among 3759 MyChart “activators” by patient characteristics (Table 2). We found that 74.1% (3759/5075) of our cohort had activated MyChart (Table 2). For age groups, 91% (111/122) of Gen Z patients had activated MyChart, whereas 69.7% (352/505) of those aged ≥75 years had activated MyChart. The rates of MyChart activation were 77.1% (2890/3749) among women and 65.5% (869/1326) among men. For race and ethnicity, 82% (2130/2586) of White patients and 87% (87/100) of Asian patients activated MyChart, whereas 63.6% (854/1342) of Black or African American patients and 65.4% (409/625) of Hispanic or Latino patients activated MyChart. MyChart activation was 76.3% (3416/4478) among English speakers and 57.4% (269/469) among Spanish speakers. The rates of MyChart activation among patients residing in a suburban county was 81.3% (1224/1506) and 65.4% (1237/1890) among patients residing in a rural county. Only 53.4% (86/161) of patients with an estimated median adjusted gross income of <US $25,000 activated MyChart, compared to 92.5% (124/134) of patients with a gross income of US $75,000 to <US $100,000. The rates of MyChart activation were high among individuals with commercial (1099/1276, 86.1%), state (319/350, 91.1%), and military (113/132, 85.6%) insurance, whereas 71.6% (1325/1851) of Medicare beneficiaries, 65.6% (336/512) of Medicaid recipients, and 65.3% (561/859) of uninsured individuals activated MyChart.

**Table 1:** Characteristics of patients—North Carolina residents with available income (N=5075).

Characteristic		Patients
**Age group (years), n (%)**		
	17-24 (Gen Z)	122 (2.4)
	25-39 (Millennials)	828 (16.3)
	40-54 (Gen X)	1538 (30.3)
	55-74 (Baby Boomers)	2082 (41)
	≥75 (Silent Gen)	505 (10)
**Sex, n (%)**		
	Female	3749 (73.9)
	Male	1326 (26.1)
**Race/ethnicity^a^, n (%)**		
	American Indian or Alaska Native or Native Hawaiian or Pacific Islander	31 (0.6)
	Asian	100 (2)
	Black or African American	1342 (26.4)
	Hispanic or Latino	625 (12.3)
	White	2586 (51)
	Other race	118 (2.3)
**Primary language^b^, n (%)**		
	English	4478 (88.2)
	Spanish	469 (9.2)
	Other	43 (0.8)
**County of residence, n (%)**		
	North Carolina urban	1679 (33.1)
	North Carolina suburban	1506 (29.7)
	North Carolina rural	1890 (37.2)
Number of visits, median (IQR)		3 (1-4)
**Median adjusted gross income (US $), n (%)**		
	<25,000	161 (3.2)
	25,000 to <50,000	3563 (70.2)
	50,000 to <75,000	1217 (24)
	75,000 to <100,000	134 (2.6)
**Insurance, n (%)**		
	Medicare	1851 (36.5)
	Commercial	1276 (25.1)
	Uninsured	859 (16.9)
	Medicaid	512 (10.1)
	State	350 (6.9)
	Military	132 (2.6)
	Department of Correction	95 (1.9)
**Primary visit diagnosis, n (%)**		
	Rheumatoid arthritis	1320 (26)
	Seronegative spondyloarthropathies	452 (8.9)
	Crystal-induced arthropathies	175 (3.4)
	Osteoarthritis and other arthropathies	524 (10.3)
	Metabolic bone diseases and other musculoskeletal conditions	517 (10.2)
	Miscellaneous inflammatory and autoimmune conditions	412 (8.1)
	Lupus and other systemic connective tissue disorders	1252 (24.7)
	Vasculitis	265 (5.2)
	Other^c^	158 (3.1)

^a^Missing race/ethnicity (n=273).

^b^Missing primary language (n=85).

^c^Nonrheumatologic conditions, nonspecific symptoms, or laboratory abnormalities.

**Table 2:** MyChart activation by patient demographics (N=3759).

Characteristic		Patient, n/N (%)
**Age group (years)**		
	17-24 (Gen Z)	111/122 (91)
	25-39 (Millennials)	670/828 (80.9)
	40-54 (Gen X)	1153/1538 (75)
	55-74 (Baby Boomers)	1473/2082 (70.7)
	≥75 (Silent Gen)	352/505 (69.7)
**Sex**		
	Female	2890/3749 (77.1)
	Male	869/1326 (65.5)
**Race/ethnicity**		
	American Indian or Alaska Native or Native Hawaiian or Pacific Islander	24/31 (77.4)
	Asian	87/100 (87)
	Black or African American	854/1342 (63.6)
	Hispanic or Latino	409/625 (65.4)
	White	2130/2586 (82.4)
	Other race	81/118 (68.6)
**Primary language**		
	English	3416/4478 (76.3)
	Spanish	269/469 (57.4)
	Other	29/43 (67.4)
**County of residence**		
	North Caroline urban	1298/1679 (77.3)
	North Carolina suburban	1224/1506 (81.3)
	North Carolina rural	1237/1890 (65.4)
**Median adjusted gross income (US $)**		
	<25,000	86/161 (53.4)
	25,000 to <50,000	2503/3563 (70.2)
	50,000 to <75,000	1046/1217 (85.9)
	75,000 to <100,000	124/134 (92.5)
**Insurance**		
	Medicare	1325/1851 (71.6)
	Commercial	1099/1276 (86.1)
	Uninsured	561/859 (65.3)
	Medicaid	336/512 (65.6)
	State	319/350 (91.1)
	Military	113/132 (85.6)
	Department of Correction	6/95 (6.3)

### MyChart Activation Pre–COVID-19

Using data from complete cases among NC residents (n=4756), we calculated the aORs of MyChart activation by patient characteristics prior to the COVID-19 pandemic (Table 3). Compared to Baby Boomers, Gen Z patients were 5 times more likely to activate MyChart (aOR 5.39, 95% CI 2.67-10.9), followed by Millennials (aOR 2.86, 95% CI 2.22-3.69) and Gen X (aOR 1.72, 95% CI 1.42-2.08) patients. Male patients were significantly less likely to activate MyChart than female patients (aoR 0.61, 95% CI 0.51-0.71; *P*<.001). Compared to White patients, Black or African American patients (aOR 0.39, 95% CI 0.33-0.47; *P*<.001) and patients of “other” race (aOR 0.44, 95% 0.27-0.70; *P*<.001) had significantly lower odds of MyChart activation. Spanish as a primary language was associated with significantly lower odds of MyChart activation (aOR 0.31, 95% CI 0.20-0.48; *P*<.001) than English. Suburban residence was associated with significantly higher odds of MyChart activation (aOR 1.22, 95% CI 1.00-1.49; *P*=.05), whereas rural residence was associated with significantly lower odds of activation (aOR 0.78, 95% CI 0.65-0.93; *P*=.007) than urban residence. Compared to patients insured through Medicare, there were significantly higher odds of MyChart activation among subjects with commercial insurance (aOR 1.77, 95% CI 1.41-2.23; *P*<.001), state insurance (aOR 2.67, 95% CI 1.76-4.05; *P*<.001), and military insurance (Tricare; aOR 2.20, 95% CI 1.19-4.10; *P*=.05), whereas Medicaid insurance was associated with significantly lower odds of MyChart activation (aOR 0.64, 95% CI 0.49-0.83; *P*<.001). Compared to thte median gross income level of US $25,000 to <US $50,000, median income of <US $25,000 was significantly associated with lower odds of MyChart activation (aOR 0.62, 95% CI 0.42-0.90; *P*=.01), whereas higher income levels were significantly associated with MyChart activation: US $50,000 to <US $75,000 (aOR 1.89, 95% CI 1.53-2.33; *P*<.001) and US $75,000 to <US $100,000 (aOR 3.61, 95% CI 1.74-7.47; *P*<.001). Results from the analysis using multiple imputed data were consistent with these results; thus, the results from complete case analyses are reported.

**Table 3:** Adjusted odds ratios (aORs) and 95% CI of MyChart activation pre–COVID-19 (n=4756; Model 1)^a^.

Characteristic			aOR (95% CI)		*P* value
**Age group (years)**					
	17-24 (Gen Z)	5.39 (2.67-10.9)		<.001	
	25-39 (Millennials)	2.86 (2.22-3.69)		<.001	
	40-54 (Gen X)	1.72 (1.42-2.08)		<.001	
	55-74 (Baby Boomers; ref)	1.00			
	≥75 (Silent Gen)	0.8 (0.62-1.03)		.08	
**Sex**					
	Female (ref)	1.00			
	Male	0.61 (0.51-0.71)		<.001	
**Race/ethnicity**					
	American Indian or Alaska Native or Native Hawaiian or Pacific Islander	1.09 (0.44-2.70)		.85	
	Asian	1.04 (0.52-2.09)		.92	
	Black or African American	0.39 (0.33-0.47)		<.001	
	Hispanic or Latino	0.86 (0.56-1.31)		.48	
	White (ref)	1.00			
	Other race	0.44 (0.27-0.70)		<.001	
**Primary language**					
	English (ref)	1.00			
	Spanish	0.31 (0.20-0.48)		<.001	
	Other	0.46 (0.21-1.01)		.05	
**County of residence**					
	North Carolina urban (ref)	1.00			
	North Carolina suburban	1.22 (1.00-1.49)		.05	
	North Carolina rural	0.78 (0.65-0.93)		.007	
**Insurance**					
	Medicare (ref)	1.00			
	Commercial	1.77 (1.41-2.23)		<.001	
	Uninsured	0.85 (0.67-1.08)		.19	
	Medicaid	0.64 (0.49-0.83)		<.001	
	State	2.67 (1.76-4.05)		<.001	
	Military	2.2 (1.19-4.10)		.05	
**Median income (US $**					
	<25,000	0.62 (0.42-0.90)		.01	
	25,000 to <50,000 (ref)	1.00			
	50,000 to <75,000	1.89 (1.53-2.33)		<.001	
	75,000 to <100,000	3.61 (1.74-7.47)		<.001	

^a^Model 1 covariables include the most recent visit date, the number of visits, age group, sex, race/ethnicity, primary language, county of residence, insurance, and median income.

### MyChart Use Pre–COVID-19 and Post–COVID-19

To determine changes in patient portal use during the COVID-19 pandemic, we calculated the aORs of MyChart usage in the 8 months prior to and the first 8 months following telemedicine adoption (Table 4). We also calculated the odds of becoming a MyChart user during the COVID-19 pandemic among those who were previously nonusers (Table 5).

Some disparities remained despite the rapid and nearly complete transition from in-person to remote care starting in April 2020 (Table 4; Model 2). The associations between MyChart use and sex, race or ethnicity, language, residency rurality, and insurance were similar to those observed with MyChart activation (Table 3; Model 1) and were not significantly different by pre–COVID-19 or post–COVID-19 timing. However, Gen Z patients had higher odds of MyChart use post–COVID-19 (aOR 2.52, 95% CI 1.63-3.89) than pre–COVID-19 (aOR 1.54, 95% CI 0.99-2.39). Interestingly, there was no difference in MyChart use after the pandemic between the highest earners (US $75,000 to <US $100,000) and the reference group (US $25,000 to <US $50,000), perhaps reflecting an increase in MyChart use among the reference group.

Among prior nonusers (n=3086; Table 5; Model 3), we observed that Gen Z was associated with significantly higher odds of becoming a MyChart user during the pandemic (aOR 2.80, 95% CI 1.32-5.94; *P*=.007). Prior male nonusers were less likely to become a MyChart user (aOR 0.58, 95% CI 0.41-0.83), as well as nonusers of rural residence compared to nonusers of urban residence (aOR 0.62, 95% CI 0.44-0.87).

**Table 4:** Adjusted odds ratios (aORs) and 95% CI of MyChart use pre–COVID-19 and post–COVID-19 (Model 2)^a,b^.

Characteristic			July 2019 to March 2020			April 2020 to November 2020		
		aOR (95% CI)		*P* value	aOR (95% CI)		*P* value	
**Age group (years)**								
	17-24 (Gen Z)	1.54 (0.99-2.39)		.05	2.52 (1.63-3.89)		<.001	
	25-39 (Millennials)	1.60 (1.30-1.97)		<.001	1.51 (1.22-1.86)		<.001	
	40-54 (Gen X)	1.37 (1.15-1.63)		<.001	1.31 (1.11-1.56)		.002	
	55-74 (Baby Boomers; ref)	1.00						
	≥75 (Silent Gen)	0.80 (0.63-1.01)		.06	0.96 (0.76-1.22)		.76	
**Sex**								
	Female (ref)	1.00						
	Male	0.81 (0.70-0.93)					.004	
**Race/ethnicity**								
	American Indian or Alaska Native or Native Hawaiian or Pacific	0.95 (0.47-1.90)					.89	
	Asian	0.75 (0.49-1.14)					.17	
	Black or African American	0.61 (0.52-0.70)					<.001	
	Hispanic or Latino	1.23 (0.90-1.69)					.19	
	White (ref)	1.00						
	Other race	0.60 (0.40-0.89)					.01	
**Primary language**								
	English (ref)	1.00						
	Spanish	0.43 (0.29-0.62)		<.001	0.55 (0.38-0.79)		.002	
	Other	1.26 (0.63-2.54)		.52	1.55 (0.78-3.09)		.21	
**County of residence**								
	North Carolina urban (ref)	1.00						
	North Carolina suburban	1.11 (0.95-1.29)					.18	
	North Carolina rural	0.80 (0.69-0.94)					.005	
**Insurance**								
	Medicare (ref)	1.00						
	Commercial	1.21 (1.02-1.45)					.03	
	Uninsured	0.67 (0.54-0.83)					<.001	
	Medicaid	0.73 (0.57-0.92)					.008	
	State	1.36 (1.05-1.75)					.02	
	Military	1.10 (0.72-1.68)					.67	
**Median income (US $)**								
	<25,000	0.70 (0.46-1.08)		.11	0.96 (0.64-1.44)		.86	
	25,000 to <50,000 (ref)	1.00						
	50,000 to <75,000	1.50 (1.27-1.77)		<.001	1.23 (1.05-1.45)		.01	
	75,000 to <100,000	1.88 (1.23-2.85)		.003	1.23 (0.82-1.84)		.32	

^a^Model additionally controls for last visit date from 2017-19 and the number of visits.

^b^Interaction terms were tested between pre–COVID-19 and post–COVID-19 time frames and all demographic covariables, and interaction terms are used to show effects by pre–COVID-19 or post–COVID-19 time frame if the interaction term was *P*<.05. Otherwise, the overall main effect is shown and is not significantly different by the pre–COVID-19 or post–COVID-19 time frame.

**Table 5:** Adjusted odds ratios (aORs) and 95% CI of MyChart use among previous nonusers (Model 3; n=3086)^a^.

Characteristic		aOR (95% CI)	*P* value
**Age group (years)**			
	17-24 (Gen Z)	2.80 (1.32-5.94)	.007
	25-39 (Millennials)	1.17 (0.75-1.81)	.49
	40-54 (Gen X)	1.20 (0.85-1.70)	.29
	55-74 (Baby Boomers; ref)	1.00	
	≥75 (Silent Gen)	0.76 (0.45-1.28)	.30
**Sex**			
	Female (ref)	1.00	
	Male	0.58 (0.41-0.83)	.003
**Race/ethnicity**			
	American Indian or Alaska Native or Native Hawaiian or Pacific	1.55 (0.34-6.99)	.57
	Asian	0.49 (0.14-1.78)	.28
	Black or African American	1.08 (0.79-1.48)	.63
	Hispanic or Latino	0.70 (0.31-1.59)	.39
	White (ref)	1.00	
	Other race	0.50 (0.17-1.48)	.21
**Primary language**			
	English (ref)	1.00	
	Spanish	1.41 (0.58-3.43)	.45
	Other	1.98 (0.38-10.2)	.41
**County of residence**			
	North Carolina urban (ref)	1.00	
	North Carolina suburban	0.97 (0.70-1.36)	.88
	North Carolina rural	0.62 (0.44-0.87)	.006
**Insurance**			
	Medicare (ref)	1.00	
	Commercial	1.00 (0.67-1.49)	.99
	Uninsured	0.76 (0.48-1.20)	.24
	Medicaid	0.73 (0.43-1.23)	.23
	State	1.29 (0.75-2.24)	.36
	Military	0.71 (0.27-1.88)	.49
**Median income (US $)**			
	<25,000	0.84 (0.37-1.88)	.67
	25,000 to <50,000 (ref)	1.00	
	50,000 to <75,000	0.95 (0.67-1.35)	.78
	75,000 to <100,000	0.34 (0.08-1.45)	.14

^a^Model additionally controls for last visit date from 2017-19 and the number of visits.

## Discussion

### Principal Findings

We found that after the start of telemedicine visits at our institution, there was a significant increase in patient portal use among our youngest patients (Gen Z generation). However, MyChart usage following the implementation of telemedicine remained significantly lower among those of Black or African American race and “other” race, having Spanish as a primary language, being uninsured, and having Medicaid. Male sex and rural residence were also associated with lower odds of MyChart use post–COVID-19, and individuals in these groups were significantly less likely to become MyChart users during the pandemic. To our knowledge, this is the first study describing disparities in patient portal use among patients with RMDs and the first to evaluate changes in health technology use during the current COVID-19 pandemic.

### Comparison With Prior Work

The results of our study build upon previous work, underscoring inequities in telehealth use and further highlighting that these disparities existed prior to the COVID-19 pandemic and continue to persist. A large cross-sectional study conducted prior to the COVID-19 pandemic showed that the most frequently cited barriers to patient portal adoption were preference for direct communication with providers and inexperience with computers, which were associated with lower income and older age. In this study, other commonly reported barriers included having no patient portal available or difficulty accessing the portal, lack of internet access, and privacy concerns [17].

Various studies examining disparities in telehealth use during the pandemic found similar results to ours. A study by Pierce and Stevermer [18] showed that those of non-White race and those who resided in rural postal codes had lower rates of telehealth use. Another study also showed that older age, living alone, and rural residence were associated with lower telehealth use [19]. However, these patterns are not restricted to rural populations. A study of New York City residents showed that Black and Hispanic patients, non-English speakers, and older patients were less likely to use telehealth for COVID-19–related care [20].

Some factors that may explain these inequities in telehealth use include limited access to affordable and reliable internet, low computer ownership, and low digital technology literacy among certain groups. It is estimated that 24 million Americans do not have access to affordable high-speed internet, with rural residents being disproportionately affected [21,22]. In a survey of NC residents, the cost and lack of access to broadband were the 2 most cited reasons for not having internet access [23]. Cellular phones offer an alternative method to access the internet and is sometimes the only option for certain individuals. A study of computer or laptop ownership among Americans showed that younger age, non-White race, lower educational attainment, and lower income were associated with “smartphone” dependency and a lack of computer or laptop ownership [24]. However, the ability to access the internet via cellular devices is subject to the availability of reliable cellular data networks.

In our study, we noted that men had lower patient portal activation and use before and during the pandemic. This finding may be explained by men’s overall lower engagement with health care and hesitancy toward help-seeking behaviors compared to women [25,26]. Similar to our findings, Yang et al [27] found that Medicaid enrollees were less likely to adopt eHealth tools compared to the non-Medicaid population in part due to lower odds of internet access.

We found that Black or African American individuals were less likely to use the patient portal even after controlling for factors such as age, insurance, residence type, and income. Thus, although there are certainly social and economic factors tied to race that contribute to lower health technology use, there seem to be other elements that influence health technology use among Black or African American individuals. Some of these results may stem from poorer access to reliable internet, privacy concerns, or preference for speaking directly to their health care providers [28,29]. A qualitative study by Lyles et al [30] evaluating barriers to patient portal use among Latinx and Black patients showed that difficulty navigating the patient portal and concern that patient portal use would diminish the patient-provider relationship were 2 major themes observed across age, income, and geographical groups. Spanish and other non-English speakers are less likely to access health care or use telehealth modalities due to difficulty communicating with providers and using health technology platforms not available in their language [31,32]. UNCH MyChart is only available in English, which prevents non–English-speaking patients from using this resource.

Age is also a large factor in telehealth use. A study investigating health technology “readiness” among older adults showed that 41.4% of Medicare beneficiaries lacked access to a computer with high-speed internet access, 40.9% lacked a smartphone with a wireless data plan, and 26.3% lacked either form of digital access [33]. Other difficulties that older adults may face include age-related impairments (eg, hearing loss, vision loss, and dementia) or low overall use and unfamiliarity with using technology, and these barriers seem to be amplified in patients who are male; single; Black or African American or Hispanic or Latino; reside in a nonmetropolitan area; and have less education, lower income, and poorer self-reported health [34].

### Strengths and Limitations

The strengths of this study include our large real-world cohort of patients with RMDs and the racial and socioeconomic diversity of our study population. We were also able to compare changes in health technology use pre–COVID-19 and post–COVID-19 in the same patients. Some of the limitations of our current study include our retrospective study design and the use of zip code as a proxy for socioeconomic variables, which risks homogenizing certain populations. Of the 5179 NC residents in our cohort, 104 were not included in the analysis due to lack of IRS income data. Of these individuals, 63% (n=66) were from rural counties, 23% (n=24) from urban counties, and 13% (n=14) from suburban counties. As expected, the majority of these individuals resided in rural counties. However, given the small percentage of the NC cohort that these patients represented (2%), excluding them from the analysis is unlikely to affect our results. We did not specifically assess remote telecommunication visits among our patients in this study and therefore cannot draw conclusions on whether there are disparities in telemedicine use among our patients. Additional factors that we did not include, but are possible confounders, include health literacy, smartphone ownership, computer or laptop ownership, broadband access, and cellular data access.

### Conclusions

As technology is increasingly used for health care delivery, addressing disparities in health technology use has never been more important. A recently published perspective piece in the New England Journal of Medicine highlighted the scope of the issue and addressed the newly enacted Infrastructure Investment and Jobs Act. This law includes funding toward broadband infrastructure development and affordability, improving connectivity in rural and tribal communities, the creation of digital literacy programs, and preventing digital discrimination [35]. Although the Infrastructure Investment and Jobs Act does not specifically address digital inequities in health care, the potential changes that may occur as a result of this law will undoubtedly affect accessibility to health technology. Although many of the issues that contribute to inequitable technology access are multifaceted and cannot be changed immediately, we hope that increased research and resources are invested in making health technology accessible for all. Future studies should focus on ongoing barriers and potential solutions in avenues such as internet accessibility, health and digital literacy, and attitudes and perceptions toward health technology.

## Abbreviations

aOR: adjusted odds ratio
IRS: Internal Revenue Service
NC: North Carolina
RMD: rheumatic and musculoskeletal disease
UNCH: University of North Carolina Hospitals
